# Supplementary figures and images for: The Candida albicans reference strain SC5314 contains a rare, dominant allele of the transcription factor Rob1 that modulates filamentation, biofilm formation, and oral commensalism
Source: mBio. 2023 Sep 22;14(5):e01521-23. doi: 10.1128/mbio.01521-23 (PMC10653842; doi:10.1128/mbio.01521-23)

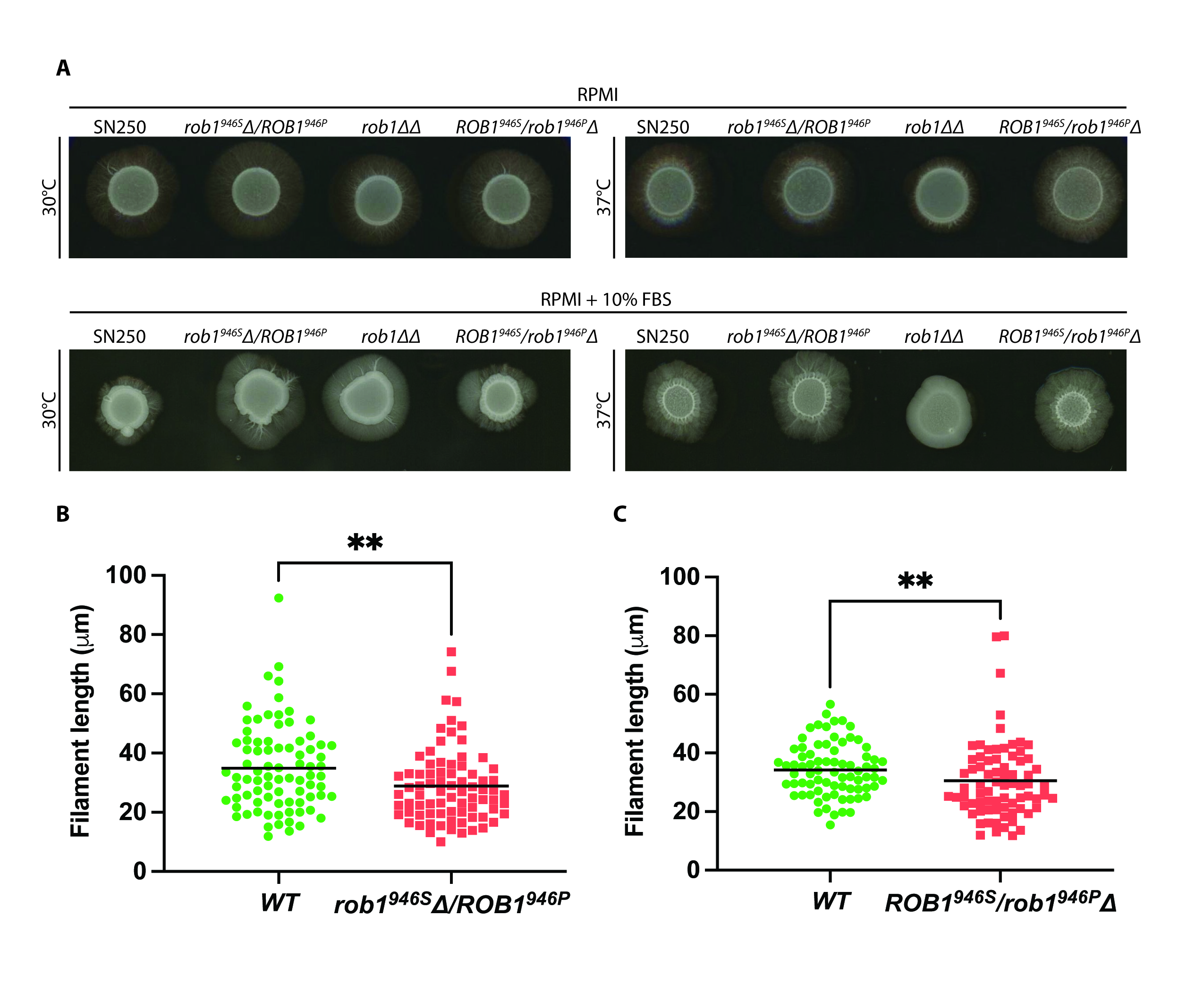

Supplement: Fig. S1 — Filamentation phenotypes of rob1 mutants. [file mbio.01521-23-s0001.tif]

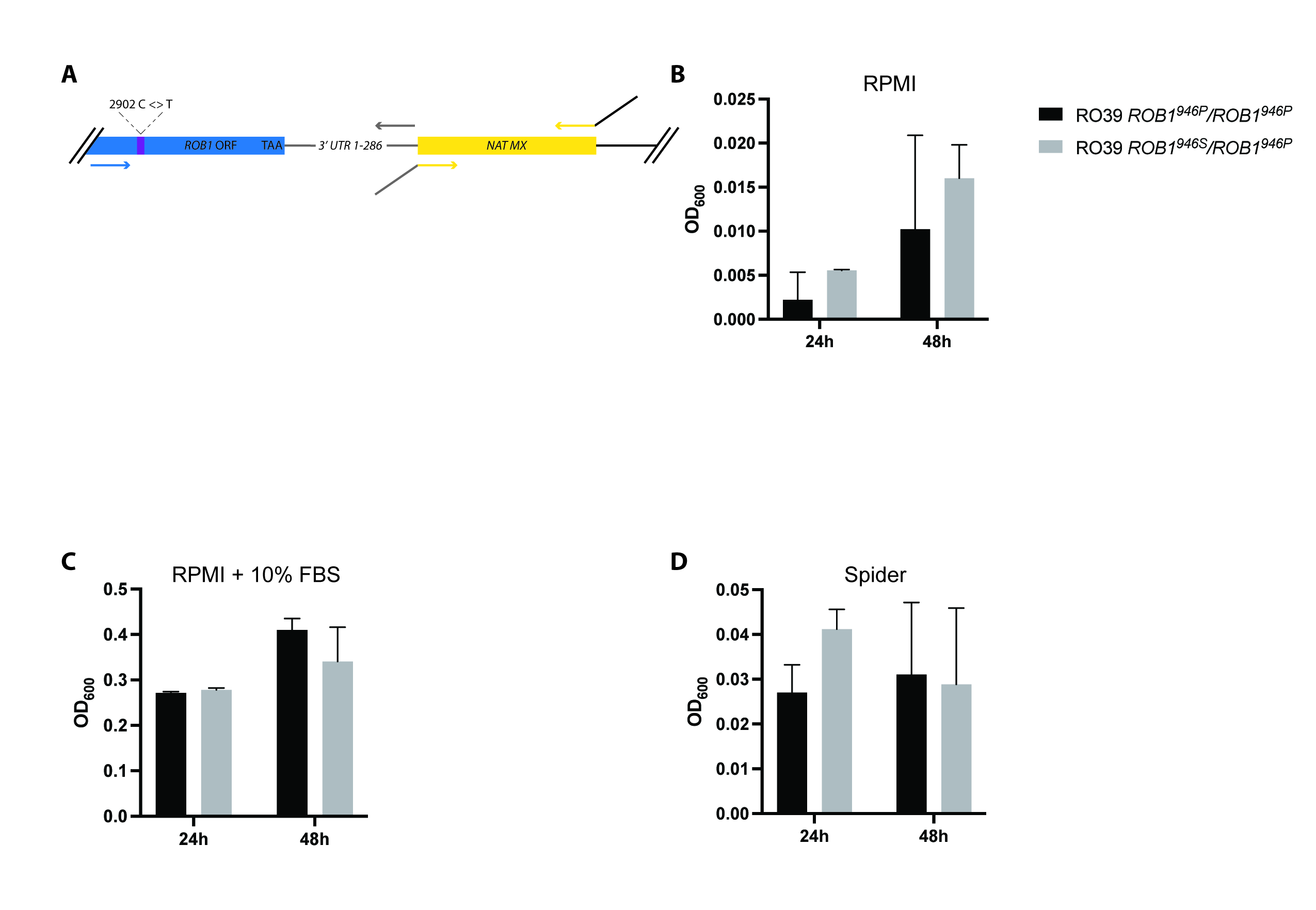

Supplement: Fig. S2 — Construction of homozygous ROB1 strains and biofilm phenotypes. [file mbio.01521-23-s0002.tif]
