# Supplementary material for: The Candida albicans reference strain SC5314 contains a rare, dominant allele of the transcription factor Rob1 that modulates filamentation, biofilm formation, and oral commensalism
Source: mBio. 2023 Sep 22;14(5):e01521-23. doi: 10.1128/mbio.01521-23 (PMC10653842; doi:10.1128/mbio.01521-23)
Supplement: Supplemental legends — Legends for supplemental figures and tables. [file mbio.01521-23-s0003.docx]

**Supplementary Material; Figure and Table legends**

**Figure S1. A.** SN250, *rob1-1*, *rob1-5*, and *rob1*∆∆ strains were spotted on RPMI and RPMI+10% BCS plates and incubated for 3 days at either 30^o^C or 37^o^C before being photographed. The images are representative of two independent experiments. **B.** The filament lengths of the *rob1-1* and *rob1-5* strains were compared to SN250 24 hr post infection as described in materials and methods. There was no difference in length as determined by Mann-Whitney U test (P<0.05).

**Figure S2 A.** Schematic of *ROB1* allele swap construct. **B**.

**Table S1. RNA sequencing data comparing the expression profile of the *rob1*∆∆ mutant to WT in Spider medium at 37^o^C after 4 hr of induction.**

**Table S2. RNA sequencing data comparing the expression profile of the *rob1*∆∆ mutant to WT in RPMI medium at 37^o^C after 4 hr of induction.**

**Table S3. Table of strains**

**Table S4. Table of oligonucleotides.**
